# Behavior‐based medical diagnosis—Applying perspectives of behavioral science to clinical reasoning

**DOI:** 10.1002/jgf2.540

**Published:** 2022-03-30

**Authors:** Kiyoshi Shikino, Yoshiyuki Ohira, Eri Sato, Akiko Ikegami, Shingo Suzuki, Kazutaka Noda, Takanori Uehara, Masatomi Ikusaka

**Affiliations:** ^1^ Department of General Medicine Chiba University Hospital Chiba‐City Chiba Japan; ^2^ Department of General Medicine International University of Health and Welfare Narita Hospital Narita‐City Chiba Japan; ^3^ Department of Internal Medicine Chiba Central Medical Center Chiba‐city Chiba Japan

**Keywords:** behavioral science, behavior‐based medical diagnosis, clinical reasoning, diagnostic error

## Abstract

Behavioral science, the scientific study of human behavior and the elucidation of its laws, is also applied to medicine, and is included in pre‐graduate education.Understanding patient behaviors that correspond to behavior‐based medical diagnosis and interpreting the clinical information suggested by these patient behaviors can be useful in avoiding diagnostic errors in clinical practice.

To the Editor,

Behavioral science, the scientific study of human behavior and the elucidation of its laws,^1^ is also applied to medicine and is included in pregraduate education.^1^ In addition, the “Behavioral and Social Science Topics of High and Medium Priority for Inclusion in Medical School Curricula” listing^2^ proposes that behavioral science topics should include mind–body interactions in health and disease, patient behavior, physician roles and behavior, physician–patient interactions, social and cultural issues in health care, and health policy and economics.

In Japan, patients can freely visit medical institutions under the national health system,^3^, ^4^ and there are unique patient behaviors that reflect this. As a result, certain variations in patients' behaviors have emerged. It is possible to apply behavioral science to clinical reasoning by understanding the variations in patient behavior. Furthermore, by incorporating the behavioral science perspective into clinical practice, it may be possible to compensate for the limitations of medical interviewing and avoid diagnostic errors.

The creation of “behavior‐based medical diagnosis,” an example of patient behavior that can be used as a clue in clinical practice, was the subject of a workshop “outpatient care using perspectives of behavioral science for avoiding diagnostic errors” at the 6th Annual Meeting of the Japanese Association for Primary Care in 2015. The workshop aimed to increase awareness of the importance of patient behavior and to develop methods to apply it to clinical practice. The participants were divided into small groups and were asked to report cases in which patient behavior provided clues to avoid diagnostic errors, and to create products by categorizing the cases. There were 54 participants (49 physicians, 2 healthcare providers, and 3 medical students). Forty cases were collected from the participants and classified into eight categories as follows (Table 1): “increase in the number of medical visits”; “seeing patients without appointments and outside hours”; “seeing a patient with a high threshold for medical care”; “unusual consultation style”; “seeing a physician in spite of minor symptoms”; “discrepancies in words and behavior”; “seeing several medical institutions for the same symptoms”; and “the purpose of the visit is for examination.” These patient behaviors suggest the following four types of clinical information: “worsening of symptoms”; “possibility of serious illness”; “focus on behavior and consider scrutiny”; and “possibly psychogenic disease.”

**TABLE 1:** Categories of behavior‐based medical diagnosis

Patient care behavior	Suggested clinical information
Increase in the number of medical visits Seeing patients without appointments and outside hours	Worsening of symptoms
Seeing a patient with a high threshold for medical care Unusual consultation style (family escort) Seeing a physician in spite of minor symptoms	Possibility of serious illness
Discrepancies in word and behavior (no symptoms, but see a physician)	Focus on behavior and consider scrutiny
Seeing several medical institutions for the same symptoms The purpose of the visit is for examination	Possibly psychogenic disease

Understanding patient behaviors that correspond to behavior‐based medical diagnosis and interpreting the clinical information suggested by these patient behaviors can be useful in avoiding diagnostic errors in clinical practice. We hope that the behavior‐based medical diagnosis strategy will help Japanese generalists.

## CONFLICT OF INTEREST

The authors have stated explicity that there no conflicts of interest in connection with this article.
